# *Amauroderma rugosum* (Blume & T. Nees) Torrend: Nutritional Composition and Antioxidant and Potential Anti-Inflammatory Properties

**DOI:** 10.1155/2013/304713

**Published:** 2013-11-25

**Authors:** Pui-Mun Chan, Gowri Kanagasabapathy, Yee-Shin Tan, Vikineswary Sabaratnam, Umah Rani Kuppusamy

**Affiliations:** ^1^Mushroom Research Centre, Faculty of Science, University of Malaya, 50603 Kuala Lumpur, Malaysia; ^2^Institute of Biological Sciences, Faculty of Science, University of Malaya, 50603 Kuala Lumpur, Malaysia; ^3^Department of Biomedical Science, Faculty of Medicine, University of Malaya, 50603 Kuala Lumpur, Malaysia

## Abstract

*Amauroderma rugosum* is a wild mushroom that is worn as a necklace by the indigenous communities in Malaysia to prevent fits and incessant crying by babies. The aim of this study was to investigate the nutritive composition and antioxidant potential and anti-inflammatory effects of *A. rugosum* extracts on LPS-stimulated RAW264.7 cells. Nutritional analysis of freeze-dried mycelia of *A. rugosum* (KUM 61131) from submerged culture indicated a predominant presence of carbohydrates, proteins, dietary fibre, phosphorus, potassium, and sodium. The ethanol crude extract (EE), its hexane (HF), ethyl acetate (EAF), and aqueous (AF) fractions of mycelia of *A. rugosum *grown in submerged culture were evaluated for antioxidant potential and anti-inflammatory effects. EAF exhibited the highest total phenolic content and the strongest antioxidant activity based on 2,2-diphenyl-1-picrylhydrazyl (DPPH) and 2,2′-azino-bis(3-ethylbenzothiazoline-6-sulphonic acid) (ABTS) assays. HF showed dose-dependent inhibition of NO production in LPS-stimulated RAW264.7 cells and NO radical scavenging activity. Gas chromatographic analysis of HF revealed the presence of ethyl linoleate and ergosterol, compounds with known anti-inflammatory properties. In conclusion, the nutritive compositions and significant antioxidant potential and anti-inflammatory effects of mycelia extracts of *A. rugosum* have the potential to serve as a therapeutic agent or adjuvant in the management of inflammatory disorders.

## 1. Introduction

Oxidative stress is caused by the imbalance between the production of reactive oxygen species (ROS) and the ability of the biological systems to detoxify reactive intermediates. This imbalance causes damage to important biomolecules and organs with potential impacts on the entire organism [1]. Most ROS are generated in cells by the mitochondrial respiratory chain, which is largely modulated by the rate of electron flow through the respiratory chain complexes. The biological reduction of molecular oxygen in aerobic cells produces ROS such as superoxide anion (O_2_^•^^−^), hydrogen peroxide (H_2_O_2_), hydroxyl radical (OH^•^), and organic peroxides, and the excess production of these radicals can oxidise and damage proteins, nucleic acids, and lipids [2]. Hence, ROS are often associated with chronic inflammation and a wide variety of cancers [3].

Inflammation is the reaction of tissue to irritation, injury, or infection, characterised by pain, redness, and swelling [4]. It is a protective process of the body that functions to destroy invading organisms or repair tissues after injury [5]. However, sustained or excessive inflammation may predispose the host to various chronic inflammatory diseases such as arthritis, asthma, multiple sclerosis, and atherosclerosis [3–5]. During inflammation, activated macrophages secrete several inflammatory mediators, including nitric oxide (NO) [6–8]. NO is synthesised by nitric oxide synthase (NOS) via oxidative deamination of L-arginine. In particular, inducible nitric oxide synthase (iNOS) catalyses the formation of a large amount of NO, which contributes to the pathogenesis of inflammatory diseases [8, 9].

Considering the undesirable side effects of anti-inflammatory drugs available in the market, natural products/herbal medicines have gained significant interest as a source of new effective therapeutic agents. Mushrooms have been consumed by humans as a component of their normal diet since ancient times. The increased interest in scientific studies on mushrooms is attributed to the significant amounts of bioactive compounds produced by the fruiting body and the mycelium liquid culture [10]. In Malaysia, selected mushrooms are used by the locals and indigenous people as a home remedy [11]. *Amauroderma rugosum* is a basidiomycete with stipe that is black with white pore surfaces that bruise to a blood red colour when touched [12]. *Amauroderma* sp., which is also known as the “epileptic child mushroom” or “cendawan budak sawan” in the Malay language, is worn around the neck by the indigenous people in Malaysia to prevent fits and incessant crying by babies [13, 14]. Fits or epilepsy has been linked with inflammation and its development is termed epileptogenesis [15]. The study of the antioxidant potential and anti-inflammatory effects of *A. rugosum *was initiated based on the traditional aboriginal belief that this wild mushroom can reduce or prevent fit episodes. To the best of our knowledge, there are no scientific reports available on the nutritional composition or antioxidant and anti-inflammatory properties of *A. rugosum*. The aim of this study was to assess the nutritive composition, antioxidant activities, and total phenolic content of mycelia extracts of *A. rugosum* grown in submerged culture and to investigate the NO radical scavenging ability and inhibitory activity of the extracts on LPS-stimulated NO production in RAW264.7 cells.

## 2. Methods

### 2.1. Chemicals

Potato dextrose broth (PDB) and potato dextrose agar (PDA) were purchased from Difco (BD, USA), ethanol was purchased from System (Selangor, Malaysia), and hexane, ethyl acetate, and dimethyl sulfoxide (DMSO) were purchased from Fisher Scientific Inc. (New Hampshire, USA). 2,2-Diphenyl-1-picrylhydrazyl (DPPH), ascorbic acid, trolox, butylated hydroxytoluene (BHT), gallic acid, Dulbecco's Modified Eagle's Medium (DMEM), foetal bovine serum (FBS), *Escherichia coli *(O55:B5) lipopolysaccharide (LPS), N_*ω*_-nitro-l-arginine-methyl ester (L-NAME), sulphanilamide, N-(1-naphty) ethylenediamine, phosphoric acid (H_3_PO_4_), quercetin, and sodium nitroprusside were obtained from Sigma-Aldrich (St. Louis, MO, USA). 2,2′-Azino-bis(3-ethylbenzothiazoline-6-sulphonic acid) (ABTS) and 3-(4,5-dimethylthiazol-2-yl)-2,5-diphenyltetrazolium bromide (MTT) were purchased from Calbiochem, Merck Millipore (Darmstadt, Germany), and potassium persulphate, Folin-Ciocalteau phenol reagent, and sodium carbonate (Na_2_CO_3_) were purchased from Merck & Co. (New Jersey, USA). Penicillin-streptomycin and fungizone were obtained from Biowest (MO, USA) and phosphate buffer saline (PBS) was purchased from Oxoid Ltd, Thermo Scientific (Hampshire, UK).

### 2.2. Mushroom Mycelia

Mycelia of *A. rugosum* (KUM 61131) were obtained from the Mycology Laboratory, Institute of Biological Sciences, University of Malaya (Kuala Lumpur, Malaysia), and maintained on PDA medium. Seven-day old mycelia grown on PDA were used as inoculum. Ten plugs from the periphery of the colony were transferred into 500 mL baffled Erlenmeyer flasks containing 100 mL of PDB medium and incubated in a shaker at 100 rpm and 27°C for 14 days. The whole broth was then freeze dried and stored at 4°C for further use.

### 2.3. Preparation of *A. rugosum* Extract

The freeze-dried mycelia broth (24.43 ± 2.12 g) was extracted with ethanol at a ratio of 1 : 10 (w/v) for two days at room temperature. The ethanolic extract was decanted and filtered using Whatman No. 4 filter paper. The extraction process was repeated five times with ethanol at a ratio of 1 : 10 (w/v), the filtrates were combined, and the excess solvent was evaporated using a rotary evaporator. The ethanol crude extract (EE; 7.74 ± 0.78 g) was subjected to further extraction with hexane at a ratio of 1 : 10 (w/v) to yield the hexane-soluble fraction (HF; 0.21 ± 0.08 g) and hexane-insoluble residue. The hexane insoluble residue was further partitioned between ethyl acetate and water (1 : 2; v/v) to yield the ethyl acetate fraction (EAF; 0.30 ± 0.08 g) and aqueous fraction (AF; 6.72 ± 1.52 g). All the extracts were stored at 4°C prior to bioassay. 

### 2.4. Nutritional Composition of Freeze-Dried Mycelia of *A. rugosum* Grown in Submerged Culture

Two hundred grams of freeze-dried mycelia of *A. rugosum* grown in submerged culture was sent to the Consolidated Laboratory (M) Sdn. Bhd. (Kuala Lumpur, Malaysia) for nutritional analysis. All tests performed were in compliance with the standards recommended by the Association of Analytical Communities/Association of Official Agricultural Chemist (AOAC) and American Association of Cereal Chemists (AACC). The cholesterol level was measured using high-performance liquid chromatography (HPLC) and the mineral contents were assessed using inductively coupled plasma optical emission spectrometry (ICP-OES).

### 2.5. Antioxidant Activity and Total Phenolic Content

#### 2.5.1. DPPH Scavenging Activity

The antioxidant activity was evaluated using DPPH, according to a modified method described by Brand-Williams et al. [16]. The DPPH was dissolved in ethanol and 195 **μ**L of this solution was added to 5 **μ**L of *A. rugosum *extract at different concentrations (0.025, 0.25, 2.5, 25, or 250 **μ**g/mL). The mixture was incubated for 3 hrs in the dark and the absorbance was measured at 515 nm using a spectrophotometer (Biotek, USA). Ascorbic acid, trolox, and BHT were used as positive controls. The values were expressed as EC_50_. EC_50_ is defined as the amount of antioxidant required to scavenge 50% of the DPPH radicals.

#### 2.5.2. ABTS Radical Scavenging Activity

The ABTS assay was used to analyse the antioxidant capacity of the *A. rugosum *mycelia extract based on the method proposed by Re et al. [17]. Briefly, ABTS radical cation (ABTS^•+^) was produced by reacting 7 mM ABTS with 2.45 mM potassium persulphate in the dark at room temperature for 12–16 hrs. The ABTS^•+^ was further diluted with ethanol to an absorbance of 0.70 (±0.02) at 734 nm and equilibrated at 30°C. One hundred microliters of ABTS^•+^ solution was added to 10 **μ**L of extract and the absorbance reading was measured after 1 min. Ascorbic acid, trolox and BHT were used as positive controls. The values were expressed as EC_50_.

#### 2.5.3. Total Phenolic Content (TPC)

Total phenolic content (TPC) was assessed based on a method reported by Cheung et al. [18] with slight modifications. Fifty microliters of 10% Folin-Ciocalteau phenol reagent was added to 50 **μ**L of extract and incubated in the dark at room temperature for 3 mins. Next, 100 **μ**L of 10% Na_2_CO_3_ was added to the mixture and the mixture was incubated in the dark at room temperature for 1 hr. The absorbance was measured at 750 nm using a spectrophotometer. Gallic acid was used as a standard phenolic compound. All determinations were carried out in triplicate and were expressed as gallic acid equivalents (GAEs). 

### 2.6. Anti-Inflammatory Potential of Extract

#### 2.6.1. Cell Culture

The murine macrophage cell line (RAW264.7 cells) from American Type Culture Collection (ATCC, CAT number TIB-71) was cultured in DMEM containing 10% FBS, 0.1% penicillin-streptomycin, 0.1% L-glutamine, and 0.1% fungizone at 37°C in a humidified atmosphere containing 5% CO_2_. When RAW264.7 cells had reached 80–90% confluency, the cells were scraped to remove them from the cell culture flask and then centrifuged at 1000 ×g at room temperature for 5 mins. The cell viability was determined by trypan blue dye exclusion method and direct counting with a hemocytometer. 

#### 2.6.2. Cell Viability

The cytotoxicity of *A. rugosum* extracts on RAW264.7 cells was determined using the MTT assay as described by Weyermann et al. [19]. RAW264.7 cells (4000 cells/well) were seeded in 96-well plates and incubated at 37°C in a humidified atmosphere containing 5% CO_2_ for 24 hrs. The attached cells were treated with *A. rugosum *extracts at different concentrations (0.01, 0.1, 1, 10, or 100 **μ**g/mL). After 24 hrs incubation, 5 mg/mL of MTT reagent was added to each well. The supernatant from each of the 96 wells containing cells was removed after 4 hrs incubation and 100% DMSO was added to dissolve the formazan salts. The viable cells reduced the pale yellow substrate to a purple formazan product. The absorbance was measured at 560 nm and the percentage of viable cells was determined relative to the control group (untreated cells). 

#### 2.6.3. Nitric Oxide Determination

The nitric oxide (NO) was determined according to the method reported by Lee et al. [20]. RAW264.7 cells (4 × 10^5^ cells/well) were seeded into 96-well plates and incubated at 37°C in a humidified atmosphere containing 5% CO_2_ for 24 hrs. The attached cells were coincubated with *A. rugosum* extracts (0.01, 0.1, 1, 10, or 100 **μ**g/mL) and 1 **μ**g/mL *Escherichia coli *(O55:B5) lipopolysaccharide (LPS) at 37°C in a humidified atmosphere containing 5% CO_2_ for another 24 hrs. The production of NO was determined by measuring the nitrite levels in the culture supernatant using Griess reagent (1% sulphanilamide and 0.1% N-(1-naphty)ethylenediamine in 2.5% H_3_PO_4_) at 540 nm. The cell viability was determined using MTT assay. N-nitro-l-arginine-methyl ester (L-NAME) at a concentration of 250 **μ**M was used as an iNOS inhibitor (positive control). A standard curve generated with sodium nitrite (0-100 **μ**M) was used to calculate the levels of nitrite produced.

### 2.7. Nitric Oxide Radical Scavenging Assay

The nitric oxide radical scavenging assay was performed according to the method described by Lee et al. [20] with slight modifications. Briefly, 10 **μ**L of *A. rugosum* extracts (0.05, 0.5, 5, 50, or 500 **μ**g/mL) was added into 96-well plates. Then, 90 **μ**L of sodium nitroprusside (5 mM dissolved in PBS) solution was added to each well and the plates were incubated for 90 mins with light exposure. Next, the Griess reagent was added to the wells and the resulting colour complex was measured at 540 nm. Quercetin was used as a positive control.

### 2.8. GC-MS Analysis

The GC-MS analysis of HF was performed with an Agilent Technologies 6890 N (United States) gas chromatography equipped with a 5975 inert mass selective detector (70 eV direct inlet) and an HP-5 ms (5% phenylmethylpolysiloxane) capillary column (30 m × 250 **μ**m × 0.25 **μ**m film thickness). The GC using helium as the carrier gas at a flow rate of 1 mL/min was initially set at 100°C, then programmed to increase to 300°C at a ramp rate of 5°C min^−1^, and was put on hold for 10 minutes at 300°C. The total ion chromatogram obtained was autointegrated by Chemstation and the constituents were identified by comparison to the mass spectral database (NIST 05 Mass Spectral Library, USA). 

### 2.9. Statistical Analysis

All values are expressed as means ± standard deviation (SD) of triplicate values. Statistical analysis was performed using one-way analysis of variance (ANOVA) followed by Duncan's Multiple Comparison Test using Statistical Product and Service Solutions, SPSS Statistics for Windows, Version 17.0, and *P* < 0.05 was denoted as being statistically significant. Effective concentrations (EC_50_) were calculated using GraphPad Prism software version 5.0. The scatter plot and regression line for the correlation between TPC and antioxidant activities were plotted using GraphPad.

## 3. Results

### 3.1. Nutritional Composition of Freeze-Dried Mycelia of *A. rugosum* Grown in Submerged Culture

The nutritional composition of freeze-dried mycelia of *A. rugosum* grown in submerged culture is depicted in Table 1. The freeze-dried mycelia grown in submerged culture contain 0.3% of the recommended daily allowance (RDA) of total fat, a nondetectable level of cholesterol, 25.5% of the RDA of carbohydrate, 16.6% of the RDA of protein, and 38.4% of the RDA of dietary fibre and are rich in minerals such as phosphorus (14.4% of the RDA), potassium (11.6% of the RDA), and sodium (25.4% of the RDA).

### 3.2. Antioxidant Activity and Total Phenolic Content

**Figure 1:**
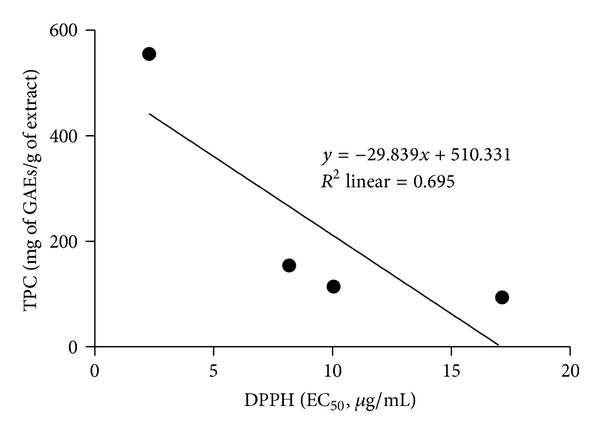
Correlation between EC_50_ value of DPPH and TPC.

**Figure 2:**
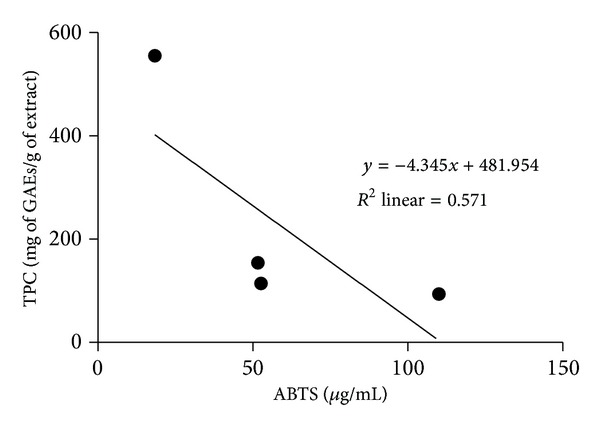
Correlation between EC_50_ value of ABTS and TPC.

The total phenolic content (TPC) of mycelia extracts of *A. rugosum *grown in submerged culture was quantified, and their antioxidant activities were evaluated based on DPPH and ABTS assays; the results are shown in Table 2. The DPPH scavenging activity of the extracts in descending order of potency was EAF > HF > AF > EE, as shown in Table 2. The descending order of the ABTS scavenging potency of the extracts was EAF > HF > AF > EE (Table 2). Besides, EAF had the highest amount of phenolic compounds, followed by HF, AF, and EE. Overall, EAF had the highest antioxidant activities and total phenolic content. The relationship between EC_50_ values of antioxidant activities and TPC is shown in Figures 1 and 2. It was found that TPC and EC_50_ values for DPPH and ABTS had a weak correlation (*R* ^2^ = 0.695 and *R* ^2^ = 0.571, *P* > 0.05, resp.).

### 3.3. The Effects of *A. rugosum* Extracts on RAW264.7 Cell Viability

**Figure 3:**
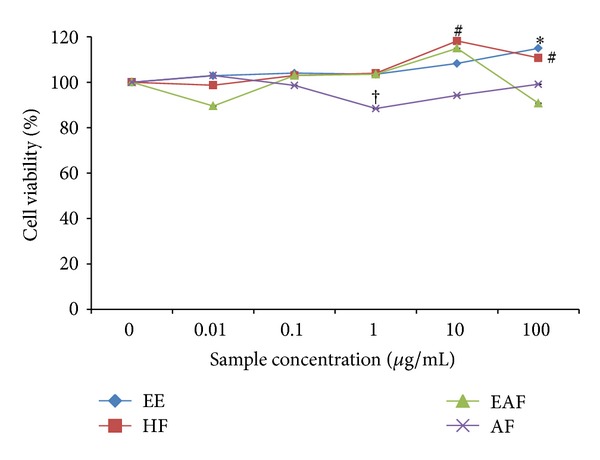
The effects of *A. rugosum *extracts on RAW264.7 cell viability. RAW264.7 cells were treated with *A. rugosum* extracts and cells without any treatment were expressed as 100%. Data were means ± S.D., *n* = 3, and ^∗, #, †^ *P* < 0.05 compared to control 100%.

The MTT assay was used to determine the effect of *A. rugosum *extracts on RAW264.7 cell viability. The cell viability of the positive control (cells without any treatment) was denoted as 100%. All the extracts tested, except for AF, had no cytotoxic effects on RAW264.7 cells (Figure 3). AF treatment at 1 **μ**g/mL caused a significant (*P* < 0.05) decrease in the number of viable cells. EE and HF promoted the proliferation of cells at concentrations greater than 1 **μ**g/mL. 

### 3.4. Inhibitory Effect of *A. rugosum *Extracts on Nitric Oxide (NO) Level in LPS-Stimulated RAW264.7 Cells

**Figure 4:**
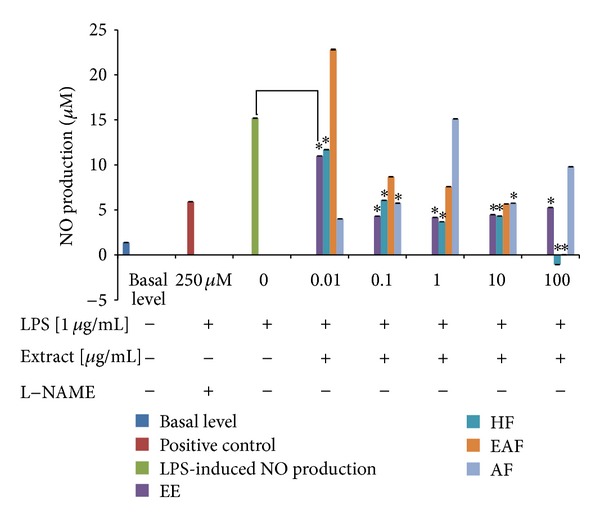
Effects of *A. rugosum *extracts on LPS-induced NO production by RAW264.7 cells. RAW264.7 cells were coincubated with various concentrations of *A. rugosum* extracts and 1 **μ**g/mL LPS for 24 hrs. L-NAME (250 **μ**M) served as positive control. Results shown represent the mean ± S.D., *n* = 3, and *P < 0.05 versus LPS-induced NO level alone.

**Figure 5:**
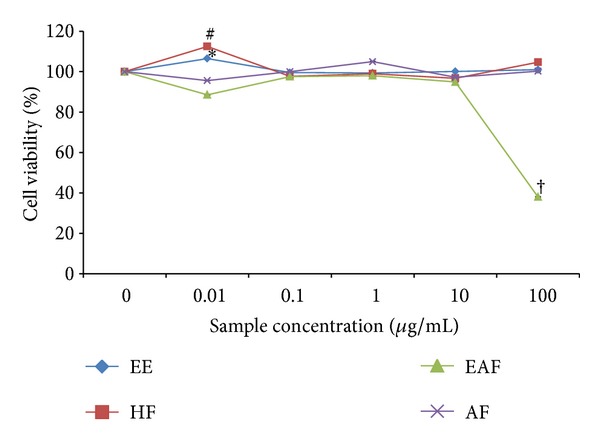
Effects of *A. rugosum* extracts on LPS-stimulated RAW264.7 cell viability. Cell viability of LPS-stimulated murine macrophage RAW264.7 cells was assessed using MTT method. Data shown were mean ± S.D., *n* = 3, and ^∗, #, †^ *P* < 0.05 compared to control 100%.

Murine macrophage RAW264.7 cells were challenged with LPS to produce NO and the effect of *A. rugosum* extracts on NO inhibition was assessed. All *A. rugosum *extracts, except for AF, inhibited NO production in a dose-dependent manner (Figure 4). The unstimulated cells secreted NO at the basal level of 1.39 ± 0.01 **μ**M, while the nontreated LPS-stimulated cells showed an increase in NO production (15.20 ± 0.01 **μ**M; 0% inhibition). L-NAME, a standard NOS inhibitor, was used as the positive control and it significantly (*P* < 0.05) inhibited NO (5.91 ± 0.01 **μ**M; 61.1%) at 250 **μ**M. Among the *A. rugosum* extracts tested, HF significantly (*P* < 0.05) inhibited NO production at all concentrations tested and inhibited it completely at 100 **μ**g/mL (Figure 4); the cell viability of HF-treated cells was 104.7% (Figure 5).

### 3.5. Nitric Oxide (NO) Radical Scavenging Activity

**Figure 6:**
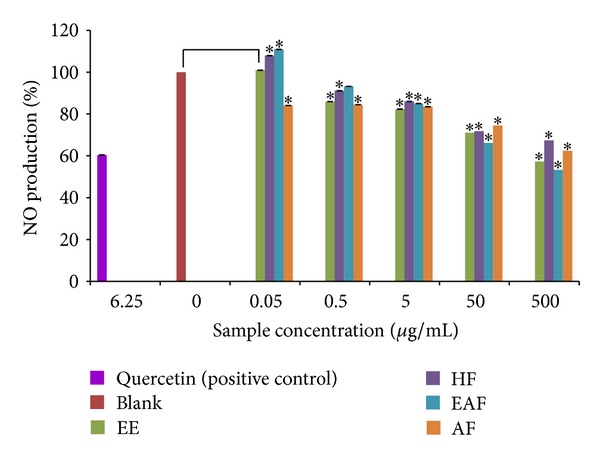
Effects of *A. rugosum* extracts on NO production by sodium nitroprusside (SNP). *A. rugosum* extracts were coincubated with SNP (5 mM dissolved in PBS) solution for 90 minutes. Quercetin (6.25 **μ**g/mL) was served as positive control. Results shown represent the mean ± S.D., *n* = 3, and *P < 0.05 versus SNP-produced NO alone.

The ability of *A. rugosum *extracts to scavenge NO radicals was evaluated. All *A. rugosum* extracts were able to scavenge NO radicals in a dose-dependent manner (Figure 6). At a concentration of 500 **μ**g/mL, EAF scavenged 46.7% of the NO radicals and this was comparable to the NO scavenging of quercetin, which was 39.6%. Quercetin is a flavonoid widely found in plants that is well known for its antioxidant and radical scavenging properties. 

### 3.6. Identification of Compounds in HF

**Figure 7:**
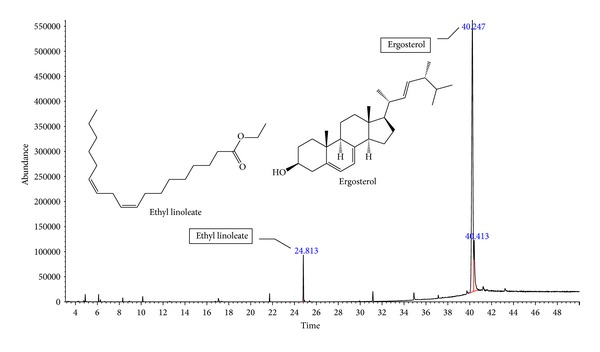
Chromatogram of HF of *A. rugosum*.

The chemical investigation of HF led to the identification of two major components, ethyl linoleate and ergosterol (Table 3, Figure 7).

## 4. Discussion

*Amauroderma rugosum* belongs to the family of Ganodermataceae and its white pore surface bruises red when touched. The sliced hollow stipe is commonly worn as a necklace around the neck by the indigenous Temuan tribe in Peninsular Malaysia to prevent fits [13]. The Temuans also believe that babies wearing the fruiting bodies as a necklace will not cry at night [14]. Epilepsy, commonly known as fits, is linked with inflammation and its development is termed epileptogenesis [15]. It has been reported that patients with refractory focal epilepsy display the hallmarks of chronic inflammation such as infiltration of leukocytes and overexpression of cytokines and targeted proteins [21]. The traditional aboriginal belief that the use of *A. rugosum* to prevent fits led to the study of the antioxidant and anti-inflammatory effects of this wild mushroom. To date, the pharmacological and biochemical activities of *A. rugosum* have not been elucidated. Hence, to the best of our knowledge, this report is the first to describe the nutritional content and medicinal properties of mycelia of *A. rugosum* grown in submerged culture.

It has been reported that mushrooms are good sources of nutritional components such as proteins, minerals, and vitamins. However, there is no existing data in the literature on the nutritional components of *A. rugosum*. The knowledge of the composition and nutritional value of wild mushrooms is limited compared to vegetables or culinary and medicinal mushrooms [22]. Thus, it is important to examine the medicinal properties and biologically active components of wild mushrooms that may benefit humankind [23]. Moreover, increasing the understanding of the ethnomedicinal use of wild mushrooms is necessary for successful bioprospecting. According to the US Food and Drug Administration (FDA), it is important to disclose the nutrient content of a dietary supplement to ensure its safety and effectiveness before and after a product is marketed. Generally, fruiting bodies of mushrooms have low fat content and high protein content (including essential amino acids) that range from 19 to 35% of the RDA and large amounts of carbohydrate and fibre, ranging from 51 to 88% and from 4 to 20% of the RDA, respectively [24]. Mhd Omar et al. [25] reported that *Lentinus squarrosulus *mycelia extract contained 4.1% carbohydrate, 0.8% total fat, and <0.1 g of the 100 g RDA of crude fibre. The present study showed that the mycelia of *A. rugosum *grown in submerged culture contained 6.2 times more carbohydrate and 96 times more fibre content than the *L. squarrosulus* mycelia extract. Furthermore, the mycelia of *A. rugosum* grown in submerged culture have lower total fat than the *L. squarrosulus* mycelia and a nondetectable level of cholesterol and are rich in minerals, such as phosphorus, potassium, and sodium. Low total fat and cholesterol content are recommended as nutritional supplements for heart patients. Also, high levels of potassium help the body process sodium, which lowers blood pressure. Furthermore, phosphorus is one of the important minerals that combines with calcium to form calcium phosphate, which gives strength and rigidity to bones and teeth.

Reactive oxygen species (ROS), the most common form of free radicals, are produced during the normal metabolism of aerobic cells. Most of the free radicals produced are neutralised by cellular antioxidant defences (enzymatic and nonenzymatic systems), and the maintenance of equilibrium between the ROS generation and neutralisation systems is essential for the normal functions of an organism [10]. Thus, disequilibrium of this physiological process causes deleterious effects in living systems and eventually causes oxidative stress. Many studies have been carried out to determine effective ways of avoiding the onset of diseases that are caused by oxidative stress. The most effective way to combat oxidative stress is by supplying the body with a greater amount of natural antioxidants. Natural antioxidants are safe, cheap, and bioactive [26]. For this reason, extensive research has been conducted to identify natural products with antioxidant aptitude that may be used for human consumption or purified into drugs. In the current study, we have found that EAF had the highest total phenolic content and antioxidant activity of all the extracts tested. This is similar to the study reported by Öztürk et al. [27], which showed that the ethyl acetate extract of *Agaricus bitorquis* had the highest total phenolic content of all the extracts of that species. The significantly higher phenolic content of EAF than crude EE is most likely due to the concentration of the phenolic compounds in the fractionation process. Moreover, the high phenolic content in EAF may contribute to its antioxidant activity [28]. Mhd Omar et al. [25] reported that the aqueous extract of *L. squarrosulus* mycelia exhibited DPPH scavenging activity with an IC_50_ value of 14.29 mg/mL. Furthermore, the DPPH scavenging activity of the ethanol extract and hot aqueous extract of *Pleurotus ferulae *mycelium showed EC_50_ values of 12.0 mg/mL and 4.3 mg/mL, respectively [29]. According to Carvajal et al. [30], the DPPH scavenging activity of the *Agaricus brasiliensis *fruiting bodies was better than the *A. brasiliensis *mycelium, but the ABTS cation radical scavenging ability analysis indicated that the mycelia had greater antioxidant activity than the fruiting bodies. Carvajal et al. [30] suggested that the discrepancy of these results may be attributed to the difference in the types of polyphenols and nonphenolic components with antioxidant activity that are present in the various extracts.

Analysis of the relationship between the TPC and antioxidant activities of *A. rugosum* extracts revealed that there was no significant correlation between them. According to Prior et al. [31], the Folin-Ciocalteau assay is used to estimate the TPC present in the extract, but free radical scavenging assays are not specific for polyphenols. Various phenolic compounds respond differently to the DPPH and ABTS assays, and the results of these assays depend on the number of phenolic groups present in the extracts [32]. Therefore, the insignificant correlation between the TPC and antioxidant activity may be attributed to the presence of nonphenolic compounds with antioxidant activities [33].

In the present study, the MTT assay was used to evaluate the cytotoxic effect of *A. rugosum* extracts on RAW264.7 murine macrophage cells. It was found that *A. rugosum *extracts had no cytotoxic effects on RAW264.7 cells, except for the AF at a concentration of 1 **μ**g/mL, which showed a significant (*P* < 0.05) but mild toxic effect. Moreover, the EE and HF significantly (*P* < 0.05) promoted proliferation of RAW264.7 cells at a higher concentration. During inflammation, numerous inflammatory mediators such as NO, cytokines, and prostaglandin E_2_ are produced [20]. However, excessive production of NO is harmful to living organisms. In the present study, the abilities of *A. rugosum* extracts to scavenge NO radicals and inhibit NO production in RAW264.7 murine macrophage cells *in vitro* were evaluated. It was found that all the *A. rugosum* extracts were able to scavenge NO radicals and reduce LPS-induced NO production in macrophages. Among the *A. rugosum *extracts tested, the EAF and HF completely inhibited NO production at a concentration of 100 **μ**g/mL. To determine whether the inhibition of NO was due to cell death or the downregulation of iNOS expression, the MTT assay was performed after challenging the cells with LPS. It was found that the reduced NO level caused by EAF at 100 **μ**g/mL was due to cell death. On the other hand, when treated with HF at 100 **μ**g/mL, RAW264.7 cells remained viable, and their NO production was inhibited. Additionally, the HF was able to scavenge NO radicals in a dose-dependent manner. Jedinak et al. [34] showed that oyster mushroom concentrate (*Pleurotus ostreatus*) markedly suppressed NO production in LPS-stimulated RAW264.7 cells at the highest concentration tested, which was 100 **μ**g/mL. Lee et al. [20] also reported that the curcumin derivative, 2,6-bis(2,5-dimethoxybenzylidene)-cyclohexanone, significantly inhibited NO production in LPS-stimulated RAW264.7 cells with an IC_50_ of 13.66 ± 0.61 **μ**M. However, the same report indicated that the curcumin derivative did not scavenge NO radicals at any concentrations tested. The HF was able to scavenge NO radicals and inhibit NO production effectively which may be an added value of this extract as a potential anti-inflammatory agent. Therefore, HF was chosen for further identification of potential compounds that may be responsible for its anti-inflammatory activities. 

Two major components were identified in HF, namely, ethyl linoleate and ergosterol. Ethyl linoleate has been reported to alleviate inflammation and its combination with antioxidants such as *α*-1-histidine, *α*-tocopherol, and tertiary butyl hydroquinone (TBHQ) can be used as a nonsteroidal topical agent [35]. Ergosterol is abundant in many mushrooms and is known to be provitamin D_2_ [36]. The conversion of ergosterol to vitamin D_2_ via UV radiation has a long history of commercial use for vitamin D_2_ production for dietary supplements, pharmaceutical grade vitamin D preparations, and food fortification [37]. A few studies have reported the successful conversion of ergosterol to vitamin D_2_ in mushrooms via UV irradiation [38, 39]. According to Ma et al. [40], ergosterol isolated from *Inonotus obliquus* was found to possess anti-inflammatory activities. Also, ergosterol purified from *Sarcodon aspratus* showed significant inhibition of LPS-induced inflammatory responses through nuclear factor-kappa B (NF-*κ*B) and CAAT/enhancer binding protein-beta (C/EBP**β**) and prevented the phosphorylation of p39, JNK, and ERK MAPKs [36].

Many studies have demonstrated that the NO inhibitory effect in RAW264.7 cells is due to the downregulation of iNOS. Inducible nitric oxide synthase (iNOS) is a soluble enzyme that catalyses the production of NO at not only the transcriptional level but also the posttranscriptional, translational, and posttranslational levels. The regulation of this pathway is important to ensure the maintenance of NO level at a constant level in living organisms. However, NO can be overproduced due to the overprotective effect of iNOS on microbial and viral pathogens. The generation of high concentrations of NO through the activation of iNOS by immunostimulating cytokines or bacterial pathogens and the activation of inducible nuclear factors such as NF-*κ*B may predispose an individual to inflammatory diseases, such as atherosclerosis, rheumatoid arthritis, diabetes, septic shock, and multiple sclerosis [41]. Thus, the inhibition of iNOS exerts a beneficial anti-inflammatory effect on inflammatory disorders. This study demonstrated that the HF significantly (*P* < 0.05) inhibited NO production in LPS-stimulated murine macrophage cells and scavenged NO radicals effectively. Although the HF did not exhibit the highest antioxidant activity compared to the other extracts tested, it was able to scavenge the DPPH radical and inhibit the ABTS^•+^ radical cation with EC_50_ values of 8.18 **μ**g/mL and 51.63 **μ**g/mL, respectively. These results suggest that EAF has strong antioxidant activity, and HF has possible roles as an antioxidant with anti-inflammatory properties.

## 5. Conclusions

In conclusion, the mycelia of *Amauroderma rugosum *are a good source of nutrients and possessed significant antioxidant and anti-inflammatory activities. Hexane fraction (HF) showed antioxidant activity and this concurred with the scavenging of NO and inhibition of NO production in LPS-stimulated macrophages (RAW264.7 cells). Ethyl linoleate and ergosterol, the two major components detected in the HF, may contribute to the activities reported. In summary, this wild mushroom, traditionally used to control epileptic episodes in children, may have potential in the mitigation of inflammatory disorders leading to epilepsy. However, further analysis of mechanisms of activity including gene expression analysis is warranted.

## Figures and Tables

**Table 1:** Nutritional analysis of freeze-dried mycelia of *A.  rugosum *grown in submerged culture.

Component	Method	Composition/100 g	Recommended daily allowance (RDA)
Energy	AOAC	341 kcal	—
Total fat	AOAC 989.05	0.2 g	65 g
Carbohydrate	AOAC	76.5 g	300 g
Protein	AACC 46-12	8.3 g	50 g
Cholesterol	HPLC	ND*	300 mg
Dietary fibre	AOAC 985.29	9.6 g	25 g
Magnesium	ICP-OES	5.69 mg	400 mg
Iron	ICP-OES	1.00 mg	18 mg
Zinc	ICP-OES	0.77 mg	15 mg
Phosphorus	ICP-OES	144.43 mg	1000 mg
Potassium	ICP-OES	404.86 mg	3500 mg
Copper	ICP-OES	0.01 mg***	2.0 mg
Manganese	ICP-OES	0.03 mg	2.0 mg
Selenium	ICP-OES	ND**	70 *μ*g
Sodium	ICP-OES	609.90 mg	2400 mg
Calcium	ICP-OES	4.66 mg	1000 mg

ND: not detectable; AOAC: Association of Analytical Communities/Association of Official Agricultural Chemist; AACC: American Association of Cereal Chemists; HPLC: high-performance liquid chromatography; ICP-OES: inductively coupled plasma optical emission spectrometry. *Level of cholesterol <0.001 mg/100 g; **level of selenium <0.02 mg/kg; ***per kg.

**Table 2:** The antioxidant activities and total phenolic content of *A. rugosum *extracts.

	Total phenolic content (mg of GAEs/g of extract)	DPPH (EC_50_ *μ*g/mL)	ABTS (EC_50_ *μ*g/mL)
Ascorbic acid	—	2.84	6.43
BHT	—	6.84	18.99
Trolox	—	4.23	17.87
EE	93.62 ± 0.01	17.14	110.00
HF	154.11 ± 0.01	8.18	51.63
EAF	555.42 ± 0.01	2.30	18.34
AF	114.14 ± 0.00	10.05	52.59

DPPH and ABTS are expressed as half maximal effective concentrations (EC_50_); TPC results are expressed as mean ± standard deviation (*n* = 3).

**Table 3:** Chemical constituents of HF.

Chemical constituents	RT (min)	MW	MF	Peak area (%)
Ethyl linoleate	24.811	308.27	C_20_H_36_O_2_	4.30
Ergosterol	40.250	396.65	C_28_H_44_O	83.79

RT: retention time; MW: molecular weight (g mol^−1^); MF: molecular formula.
